# Sacubitril/valsartan mitigates cardiac remodeling, systolic dysfunction, and preserves mitochondrial quality in a rat model of mitral regurgitation

**DOI:** 10.1038/s41598-023-38694-6

**Published:** 2023-07-16

**Authors:** Lalida Tantisuwat, Nakkawee Saengklub, Pakit Boonpala, Sarawut Kumphune, Yaowalak Panyasing, Sarinee Kalandakanond-Thongsong, Anusak Kijtawornrat

**Affiliations:** 1 Department of Physiology, Faculty of Veterinary Science, Chulalongkorn University, Bangkok, Thailand; 2 Department of Physiology, Faculty of Pharmacy, Mahidol University, Bangkok, Thailand; 3 Biomedical Engineering Institute (BMEI), Chiang Mai University, Chiang Mai, Thailand; 4 Biomedical Engineering and Innovation Research Centre, Chiang Mai University, Chiang Mai, Thailand; 5 Department of Pathology, Faculty of Veterinary Science, Chulalongkorn University, Bangkok, Thailand; 6 Chulalongkorn University Laboratory Animal Center (CULAC), Chulalongkorn University, Bangkok, Thailand

**Keywords:** Cardiovascular biology, Cardiology, Pharmacology

## Abstract

Sacubitril/valsartan (SAC/VAL), an angiotensin receptor blocker-neprilysin inhibitor, has been widely used to treat several types of heart failure. Nevertheless, the effects of drugs in mitral regurgitation patients, from the molecular level to therapeutic effects, remain unclear. This study investigates the roles of SAC/VAL on cardiac function, mitochondrial quality, autophagy, mitophagy, and natriuretic peptides in a rat model of chronic mitral regurgitation. Male Sprague–Dawley rats underwent MR induction (n = 16) and sham surgeries (n = 8). Four weeks post-surgery confirmed MR rats were randomly divided into MR (n = 8) and SAC/VAL (n = 8) groups. The SAC/VAL group was administered SAC/VAL, whereas the MR and the sham rats received vehicle via oral gavage daily for 8 weeks. Cardiac geometry, function, and myocardial fibrosis were assessed by echocardiography and histopathology. Spectrophotometry and real-time PCR were performed to assess the pharmacological effects on mitochondrial quality, autophagy, mitophagy, and natriuretic peptides. MR rats demonstrated significant left heart dilation and left ventricular systolic dysfunction compared with the sham group, which could be significantly improved by SAC/VAL. In addition, SAC/VAL significantly reduced myocardial cardiac remodeling and fibrosis in MR rats. SAC/VAL improved the mitochondrial quality by attenuating mitochondrial reactive oxygen species production and mitochondrial depolarization compared with the MR group. Also, the upregulation of autophagy-related, mitophagy-related, and natriuretic peptide system gene expression in MR rats was attenuated by SAC/VAL treatment. In conclusion, this study demonstrated that SAC/VAL treatment could provide numerous beneficial effects in MR conditions, suggesting that this drug may be an effective treatment for MR.

## Introduction

Mitral regurgitation is the most prevalent valvular disease in the aging population^1^, and the most common heart disease found in small-breed, middle-old aged dogs^2^. The renin–angiotensin–aldosterone system (RAAS) and natriuretic peptide system (NPs) are essential mechanisms that are accelerated as compensatory mechanisms to preserve cardiac output and stabilize haemodynamic status when mitral regurgitation occurs along with its consequences, including left ventricular (LV) volume overload, LV systolic dysfunction, and LV remodeling^3,4^. However, if MR progression continuously develops with chronic activation of these systems, they will become a vicious cycle, culminating in subsequent heart failure (HF)^5^. HF is a necessary consequence of MR, resulting in a high and increasing mortality rate worldwide^6^.

Recently, studies have mentioned several cellular mechanisms contributing to the disease progression through HF. Volume overload and RAAS activation are critical factors that initiate reactive oxygen species (ROS) production, inducing mitochondrial damage and dysfunction and leading to cardiac injury, remodeling, and HF^7^. During this period, autophagy upregulation is responsible for dysfunctional protein turnover and cell biogenesis preservation^8,9^. Microtubule-associated protein 1A/1B-light chain 3 (LC3), an indicator of autophagy, is upregulated in MR^10^ and was significantly associated with the severity of the myolysis in MR^11^. According to this point of view, novel pharmacological interventions for HF patients are continuously being developed that focus deeper on mechanisms involved with disease pathophysiology^12^. The drugs that can positively affect these mechanisms will be effective treatments for cardiac patients in the long run.

Sacubitril/valsartan (SAC/VAL) combines an angiotensin II type 1 receptor (AT_1_R) blocker and a neprilysin inhibitor, ARNi, was approved by the U.S. Food and Drug Administration for heart failure (HF) with reduced ejection fraction treatment in 2015^13^. The dual mechanisms of SAC/VAL are to inhibit the over-activation of pathological RAAS through an AT_1_R blocker and enhance the salutary effects of NPs by degrading the neprilysin enzyme and having potentially synergistic effects^14^. Previous studies revealed that SAC/VAL improved cardiac function and limited cardiac remodeling, inflammation, and fibrosis in experimental and clinical studies of cardiovascular diseases^14–17^. Clinical studies in HF patients reported that the drugs significantly reduced hospitalization and mortality rates^18^ and mitigated regurgitant volume in patients with secondary functional MR^19^. Also, growing evidence supports that SAC/VAL has beneficial effects on attenuating ROS production, mitigating mitochondrial dysfunction, and autophagy deactivation in doxorubicin-induced cardiomyopathy mice, pressure overload rats, and cardiorenal syndrome rats^17,20,21^.

Nonetheless, the effects of SAC/VAL on cardiac function, cardiac remodeling, and fibrosis in MR conditions have not been thoroughly investigated. In addition, the effects of SAC/VAL on mitochondrial quality and autophagy/mitophagy mechanisms in MR have yet to be elucidated. Accordingly, this study aims to evaluate the potential effects of SAC/VAL on cardiac geometry, cardiac function, mitochondrial quality, and autophagy in a rat model with chronic MR.

## Results

### Cardiac function after mitral regurgitation

Surgery-induced MR was performed by the posterior mitral leaflet puncture in rats to evaluate whether SAC/VAL attenuates left heart enlargement secondary to MR and preserved LV function. Then, drug administration was performed 4 weeks after surgery and continued daily for 8 weeks. At the end of 8 weeks after medication, thoracic echocardiography was used to evaluate cardiac geometry and function.

**Figure 1:**
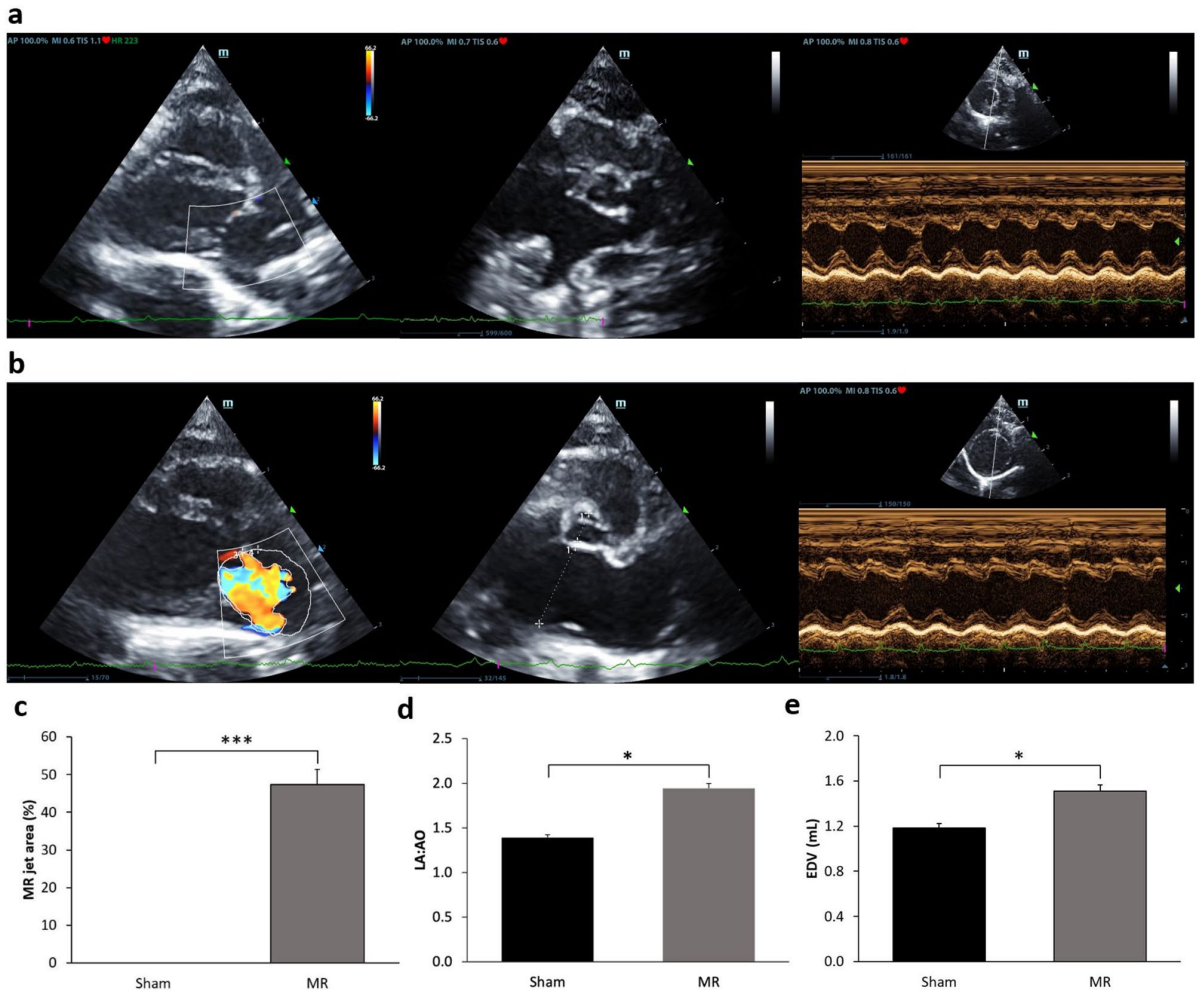
Representative echocardiography images from sham and mitral regurgitation groups at baseline (**a**, **b**). The MR group demonstrated a significant increase in the MR jet area when compared with the sham group (**c**). The MR operation significantly increased the left atrial to aortic root ratio (LA: Ao) and end-diastolic volume in MR rats when compared with the sham rats, respectively (**d**, **e**). Sham n = 8, MR n = 16. Data are presented as mean ± SEM. Statistical significance was determined using the Student *t*-test. *P < 0.05, ***P < 0.001 as indicated.

Four weeks after MR induction, significant post-MR left atrial (LA) and LV dilation were confirmed by echocardiography (Fig. 1a, b). All rats with MR demonstrated an average MR jet area of 47.31% ± 4.1%, whereas the MR jet was not exhibited in the sham rats (Fig. 1c). In addition, there was significantly marked LA dilation (Sham: 1.39 ± 0.03 vs. MR: 1.98 ± 0.05, *P* < 0.05) along with an increase in end-diastolic volume (Sham: 1.18 mL ± 0.04 mL vs. MR: 1.51 mL ± 0.06 mL, *P* < 0.05) at 4 weeks after the MR induction (Fig. 1d, e). Other parameters of LV functions were not different between the sham and the MR groups.

### Cardiac geometry and function after sacubitril/valsartan treatment

**Figure 2:**
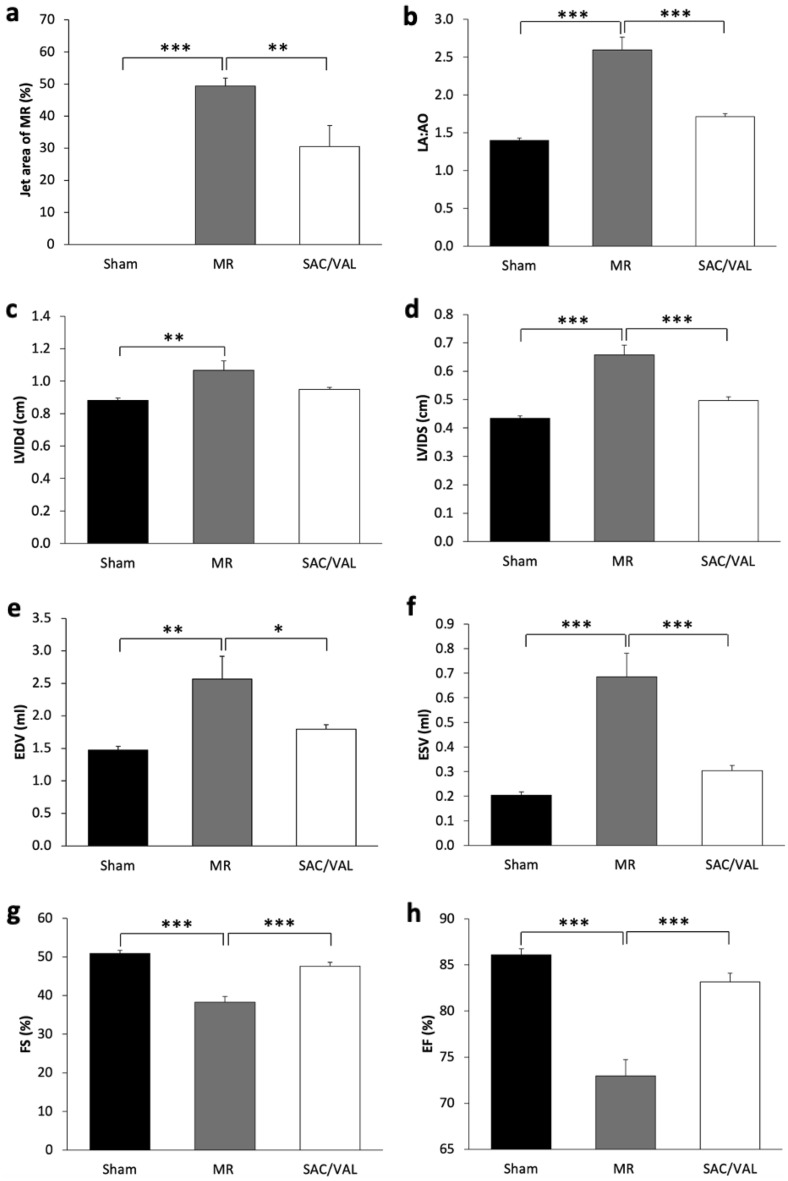
Pharmacological effects of SAC/VAL on cardiac geometry and LV systolic function. (**a**) Mitral regurgitation and sacubitril/valsartan (SAC/VAL) rats showed a significantly higher MR jet area than sham rats did. In contrast, SAC/VAL significantly decreased the MR jet area as compared with MR. (**b**) The SAC/VAL significantly decreased left atrial to aortic root ratio (LA:Ao) compared with MR. (**c**, **d**) A significant reduction of left ventricular internal diameter diastole (LVIDd) and the left ventricular internal diameter systole (LVIDs) was seen in SAC/VAL group compared with the MR group. (**e**, **f**) The end-diastolic volume and end-systolic volume (ESV) decreased significantly with SAC/VAL treatment compared with vehicle treatment. (**g**, **h**) The SAC/VAL treatment increased fractional shortening (FS) and ejection fraction (EF) compared with the vehicle-treated rats. Sham n = 8, MR n = 8, SAC/VAL n = 8. Data are presented as mean ± SEM. Statistical significance was determined by a one-way ANOVA followed by Tukey’s post-hoc for multiple comparisons. *P < 0.05, **P < 0.01, ***P < 0.001 as indicated.

MR rats demonstrated a significant augmentation of the MR jet area as compared with the sham rats (Sham: 0% vs. MR: 49.4% ± 2.45%, *P* < 0.001), whereas treatment with SAC/VAL showed a significant decrease in this parameter when compared with the MR (SAC/VAL: 30.52% ± 6.58%, *P* < 0.01) (Fig. 2a). MR rats presented a marked increase in the left atrial to aortic root ratio (LA:Ao) (Sham: 1.4 ± 0.03 vs. MR: 2.59 ± 0.17 cm, *P* < 0.001) (Fig. 2b) and left ventricular internal diameter diastole (LVIDd) (Sham: 0.88 ± 0.01 cm vs. MR: 1.07 ± 0.06 cm, *P* < 0.01) as well as left ventricular internal diameter systole (LVIDs) (Sham: 0.43 ± 0.01 cm vs. MR: 0.66 ± 0.04 cm, *P* < 0.001). SAC/VAL tended to attenuate the severity of LV dilation by reducing LVIDd (*P* = 0.064) and significantly reduced LVIDs (SAC/VAL: 0.5 ± 0.01 cm, *P* < 0.001) (Fig. 2c, d), which was accompanied by significantly blunted in the severity of LA enlargement inferred from the LA:Ao when compared with the MR group (SAC/VAL: 1.71 ± 0.04, *P* < 0.001). Consistently with decrease LV dilation, EDV (MR: 2.57 ± 0.35 mL vs. SAC/VAL: 1.8 ± 0.07 mL, *P* < 0.05) and end-systolic volume (ESV) (MR: 0.69 ± 0.1 mL vs. SAC/VAL: 0.3 ± 0.02 mL, *P* < 0.001) were significantly lower in the SAC/VAL group (Fig. 2e, f). However, LV wall thickness of both the interventricular septum and free wall during systole and diastole showed no statistically significant alteration among the groups.

A highly significant decrease of fractional shortening (FS) (Sham: 50.86% ± 0.82% vs. MR: 38.31% ± 1.48%, *P* < 0.001) was observed together with a significant decrease of ejection fraction (EF) (Sham: 86.09% ± 0.66% vs. MR: 72.97% ± 1.75%, *P* < 0.001) in the MR group when compared with the sham (Fig. 2g, h), pointing to LV systolic dysfunction. Administration of SAC/VAL significantly prevented LV systolic dysfunction by demonstrating a significantly higher FS (SAC/VAL: 47.6% ± 1.03%, *P* < 0.001) and EF (SAC/VAL: 83.17% ± 0.96%, *P* < 0.001) as compared with the MR rats (Fig. 2g, h).

### Cardiac remodeling and fibrosis

**Figure 3:**
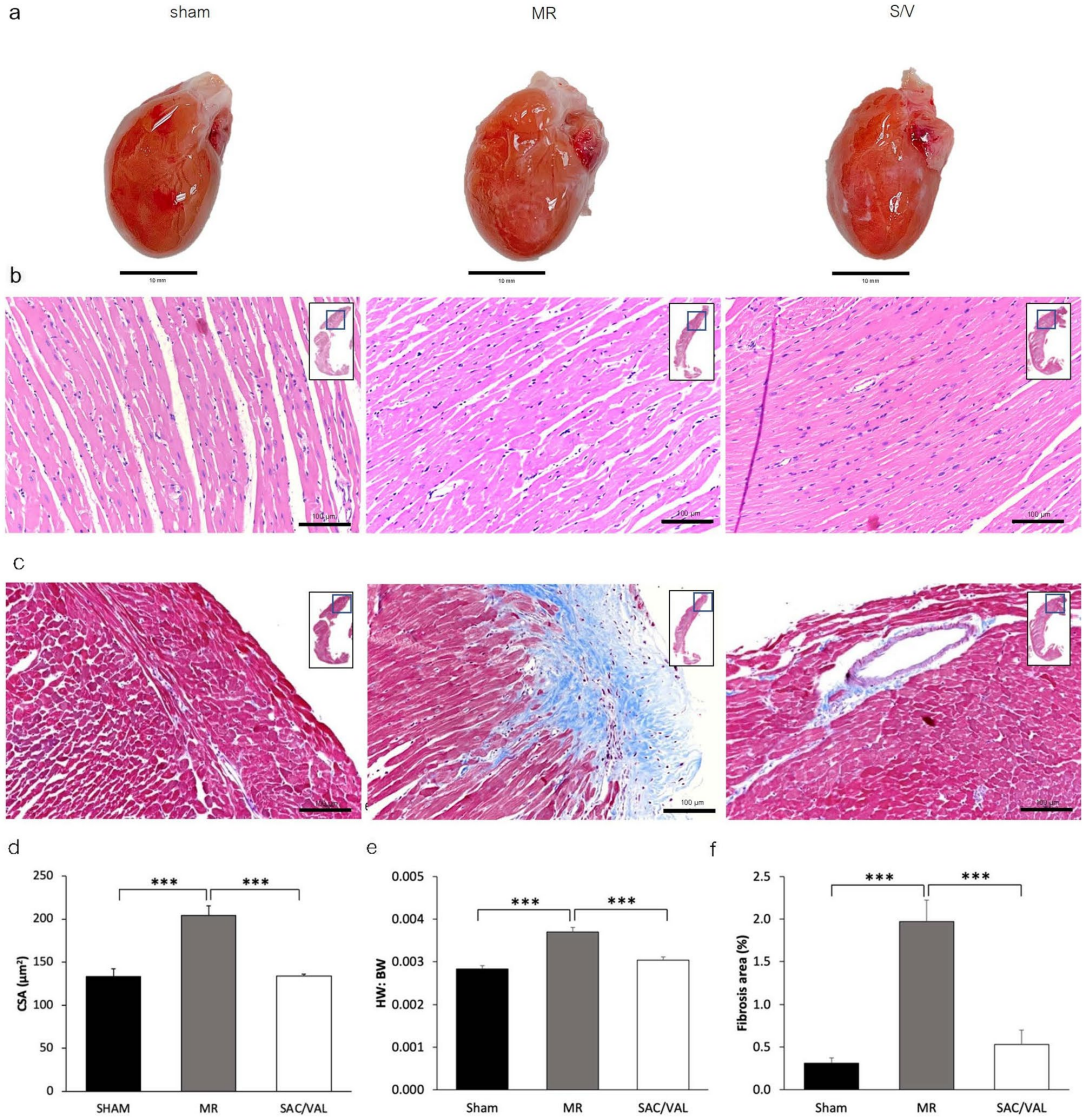
LV structural remodeling and fibrosis. (**a**) Representative images of macroscopic findings of the heart from all groups. (**b**) Representative hematoxylin and eosin (H&E) staining in the left ventricle (LV) sectioned in all study groups at the end of the study. (**c**) Representative images of LV sectioned stained with Masson’s trichrome stain in the sham, MR, and sacubitril/valsartan (SAC/VAL) rats. The fibrosis area is presented in blue. (**d**, **e**) The significant reduction in cardiac remodeling, determined by left ventricular myocyte cross-sectional area (CSA) and heart weight per body weight ratio (HW:BW), is demonstrated in the SAC/VAL administration group compared with the MR group. (**f**) Quantification of myocardial fibrosis area (%). SAC/VAL significantly limited fibrosis area compared with MR rats. Sham n = 8, MR n = 8, SAC/VAL n = 8. Fibrosis area (%) was quantified as the collagen-stained area/the entire sectioned area) × 100. Data are presented as mean ± SEM. Statistical significance was determined by a one-way ANOVA followed by Tukey’s post-hoc for multiple comparisons. ***P < 0.001 as indicated.

At the end of the study, the heart was harvested and weighed to obtain the heart weight (HW) per body weight ratio and embedded for a histopathological study. The gross morphology of the whole heart was compared among three groups (Fig. 3a). From histopathology, the descriptive findings from hematoxylin and eosin (H&E) stain analyzed by a pathologist revealed that myocardium of some animals from all groups had mild myocardial degeneration, i.e., hyaline degeneration, disorganization of cardiac myocytes and myofibrils. This morphological alteration presented in a few small areas of the myocardium; no noticeable difference was found among the groups (Sham, MR, SAC/VAL) (Fig. 3b). However, the Masson’s trichrome stain of the LV section from the MR group demonstrated a significantly larger fibrosis area than that of the sham group (Sham: 0.31% ± 0.06% vs. MR: 1.97% ± 0.25%, *P* < 0.001), which could be significantly reduced by SAC/VAL (SAC/VAL: 0.53% ± 0.17%, *P* < 0.001) (Fig. 3c, f).

The HW (Sham: 1.71 ± 0.03 g vs. MR: 2.24 ± 0.09 g, *P* < 0.001), left ventricular myocyte cross-sectional area (CSA) (Sham: 133.5 ± 8.8 µm^2^ vs. MR: 204.0 ± 11.2 µm^2^, *P* < 0.001), and the HW:BW (Sham = 0.0028 ± 0.0001 g vs. MR: 0.0037 ± 0.0001 g, *P* < 0.001) demonstrated a significant left heart remodeling in the MR group when compared with the sham group. The SAC/VAL significantly mitigated cardiac remodelling inferred from the heart weight (SAC/VAL: 1.73 ± 0.06 g, *P* < 0.001), CSA (SAC/VAL: 133.9 ± 2.4 µm^2^, *P* < 0.001), and HW: BW (SAC/VAL: 0.003 ± 0.0001 g, *P* < 0.001) (Fig. 3d, e).

### Mitochondrial quality

**Figure 4:**
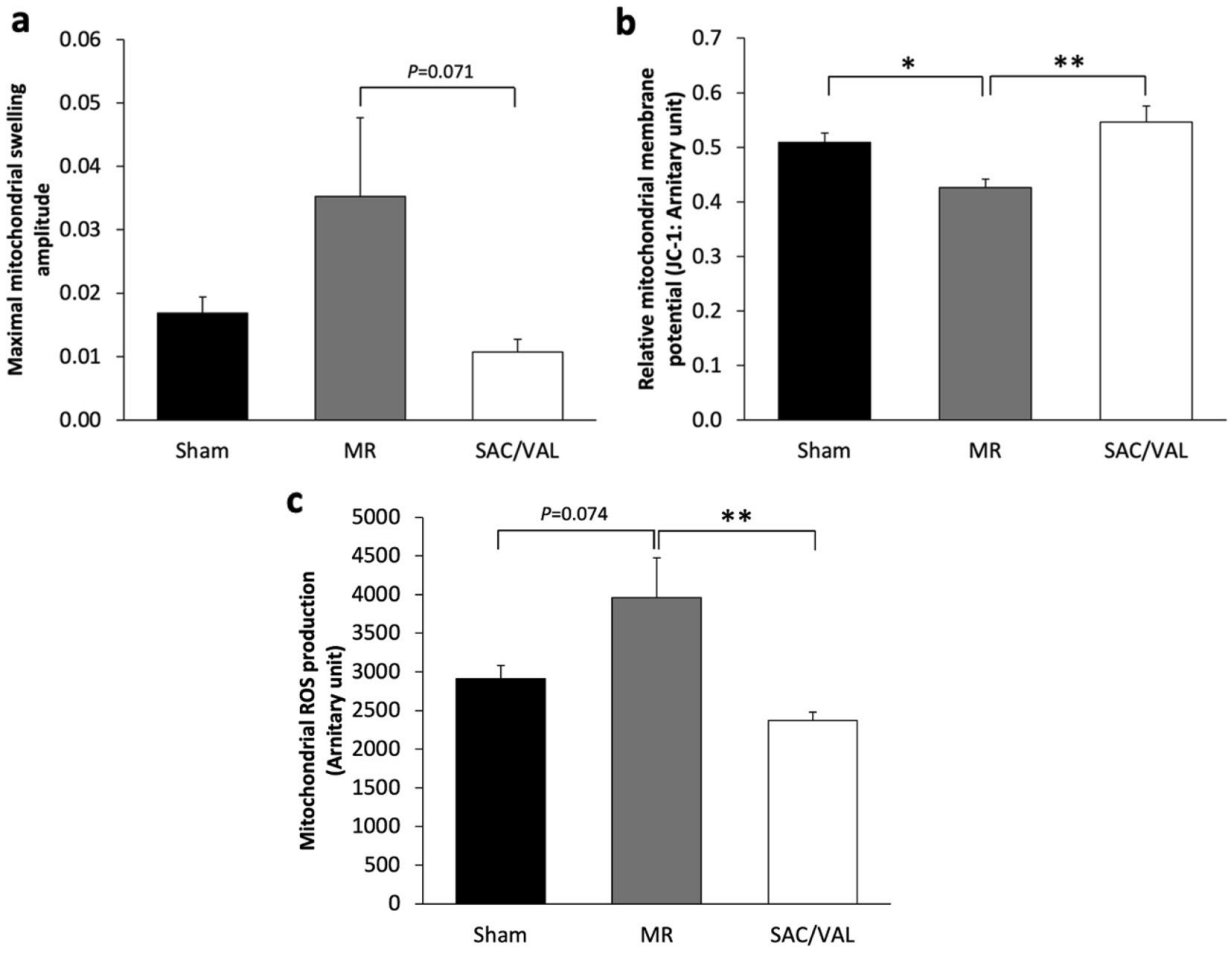
Mitochondrial quality analysis at the end of 8 weeks of drug administration. (**a**) Sacubitril/valsartan (SAC/VAL) revealed a trend to decrease mitochondrial swelling compared with mitral regurgitation. (**b**) SAC/VAL significantly prevented the mitochondrial membrane potential change caused by MR. (**c**) SAC/VAL treatment significantly diminished mitochondrial reactive oxygen species (ROS) production when compared with vehicle-treated rats. Sham n = 8, MR n = 8, SAC/VAL n = 8. Data are presented as mean ± SEM. Statistical significance was determined by a one-way ANOVA followed by Tukey’s post-hoc for multiple comparisons. *P < 0.05, **P < 0.01 as indicated.

Mitochondrial quality assessments were performed using spectrophotometry. The maximal mitochondrial swelling amplitude did not differ between the sham and the MR groups, whereas SAC/VAL tended to decrease in this parameter (MR: 0.035 ± 0.01 vs. SAC/VAL: 0.011 ± 0.002, *P* = 0.071) (Fig. 4a). The disease condition had a marked decrease in relative mitochondrial membrane potential (Sham: 0.51 ± 0.02 vs. MR: 0.43 ± 0.02, *P* < 0.05) (Fig. 4b) and a trend to increase mitochondrial ROS production (Sham: 2913 ± 169 vs. MR: 3961 ± 514, *P* = 0.074) (Fig. 4c). Interestingly, when compared with MR rats, SAC/VAL significantly preserved and enhanced mitochondrial quality by maintaining mitochondrial membrane potential (SAC/VAL: 0.546 ± 0.03, *P* < 0.01). Moreover, SAC/VAL administration of significantly attenuated cardiac mitochondrial ROS production caused by MR (SAC/VAL: 2372 ± 110, *P* < 0.01).

### Autophagy–mitophagy-related gene expression and natriuretic peptide gene expression

**Figure 5:**
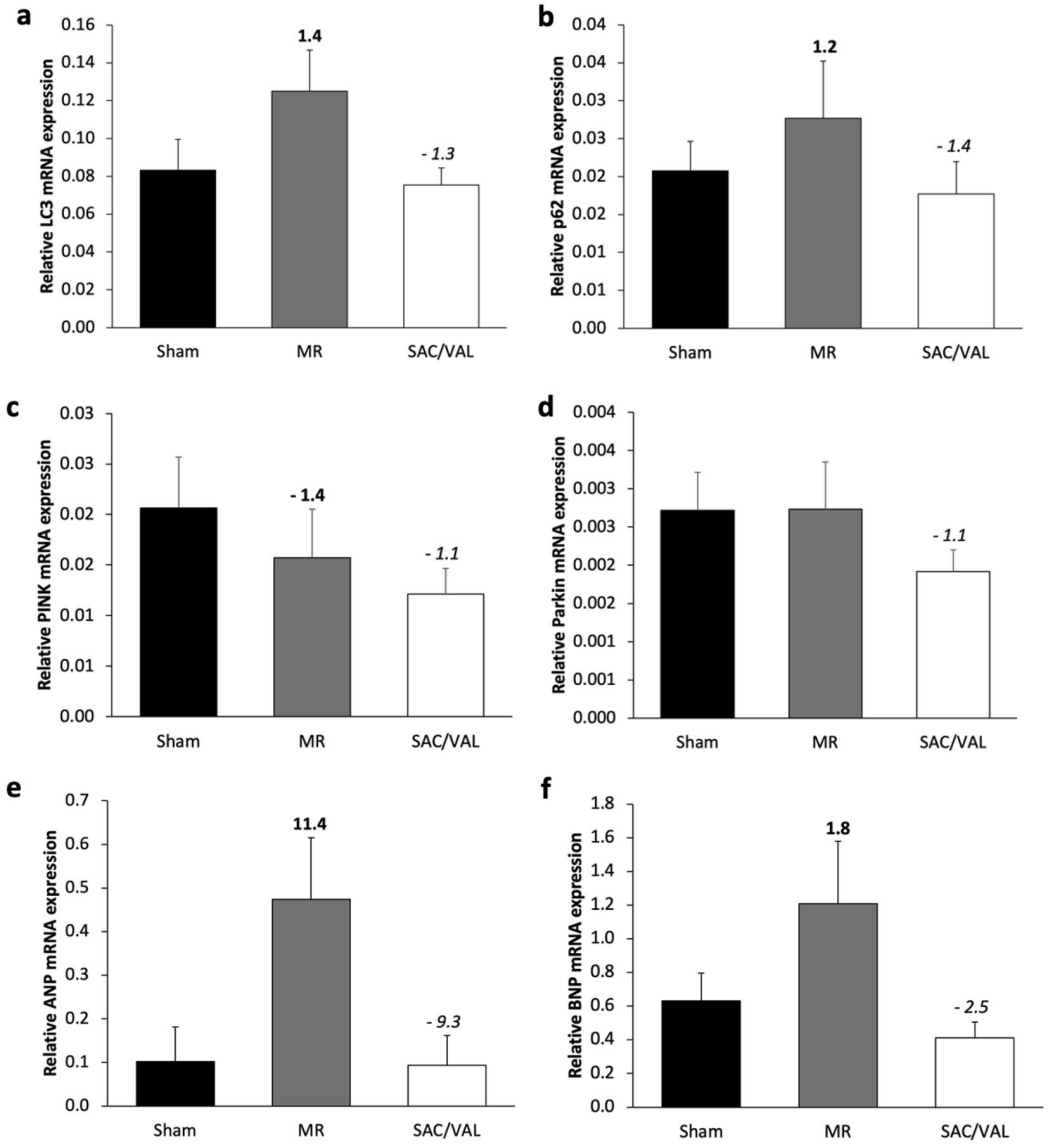
Expression of autophagy-related genes, mitophagy-related genes, and NPs-related genes at the end of the study. (**a**, **b**) Sacubitril/valsartan (SAC/VAL) significantly attenuated the upregulation of autophagy-associated genes (LC3 and p62) in mitral regurgitation rats. (**c**, **d**) There was a slight downregulation in mitophagy-related genes (PINK and Parkin) due to the SAC/VAL treatment. (**e**, **f**) The upregulation of atrial natriuretic peptide (ANP) and brain (B-type) natriuretic peptide (BNP) mRNA expression levels were demonstrated in the SAC/VAL group compared with the MR group. Data are presented as mean ± SEM. Sham n = 8, MR n = 8, S/V n = 8. Numbers in bold indicate the fold change from sham, whereas numbers in italics indicate the fold change from MR.

The mRNA expression of autophagy and mitophagy-related genes in the LV tissue was determined using RT-PCR to determine whether SAC/VAL influences autophagy and mitophagy mechanisms. The expression of LC3 and p62 mRNA were increased 1.4-fold and 1.2-fold due to MR, respectively, indicating an upregulation of these genes in MR rats. The SAC/VAL revealed a reduction in LC3 and p62 mRNA expression by 1.3-fold and 1.4-fold (Fig. 5a, b). In addition, compared with the sham, MR reduced the expression of PTEN-induced kinase 1 (PINK1) mRNA by 1.4-fold. In contrast, there was no alteration in Parkin mRNA expression. Interestingly, SAC/VAL slightly reduced the expression of these mitophagy-related genes by 1.1-fold when compared with MR rats (Fig. 5c, d).

The pharmacological effects of SAC/VAL on the relative atrial natriuretic peptide (ANP) and brain (B-type) natriuretic peptide (BNP) mRNA expression were also investigated. The results revealed increases in ANP and BNP mRNA expression by 11.4-fold and 1.8-fold due to MR, respectively. However, the expressions of ANP and BNP mRNA were reduced 9.3-fold and 2.5-fold due to SAC/VAL treatment when compared with MR (Fig. 5e, f).

## Discussion

This study evaluated the pharmacological effects of SAC/VAL on cardiac function, cardiac remodeling, LV fibrosis, mitochondrial quality, autophagy, and NPs in rats with chronic MR. The major finding of this study revealed that SAC/VAL prevents left heart enlargement, LV systolic dysfunction, cardiac remodeling, and LV fibrosis in response to chronic MR. Furthermore, SAC/VAL maintains and preserves the mitochondrial quality and limits mitochondrial ROS production in the LV tissue from chronic MR rats. At the molecular level, the drug also attenuated the upregulation of autophagy, mitophagy, and NPs in MR-volume overload conditions. Altogether these data suggested that SAC/VAL provides several beneficial effects on mitigating various pathophysiological processes that usually lead to HF and promoting cardioprotective effects in the MR hearts.

Even though MR is a prevalent cardiac disease in humans and animals^1,2^, the effects of SAC/VAL in this disease setting remain scarce. Accordingly, we performed MR-induced surgery to mimic MR and its consequence, volume overload, in rats. This model has been developed, verified, and used in previous studies with the primary objective of evaluating the pharmacological effects of cardiovascular drugs on MR conditions^22,23^. Four weeks after MR creation has been mentioned as a chronic stage of MR in a small animal model with substantial LV dilation^22^. Therefore, we initiated SAC/VAL administration during this period.

As expected, MR vehicle-treated rats demonstrated significantly left heart enlargement compared with the sham group at the end of the study, which was evidenced by increased LA: Ao, LVIDd, LVIDs, ESV, and EDV from echocardiography. In addition, evidence of LV systolic impairment was observed during this period. SAC/VAL notably decreased LA and LV dilation resulting from chronic MR. The results agreed with prior studies in which SAC/VAL substantially attenuated LA, LV enlargement, and volume overload in functional MR patients and experimental HF mice and rats^19,24^. Besides reducing left heart dilation, SAC/VAL also preserved LV systolic function, as indicated by the significant increase in FS and EF compared with MR. These findings are consistent with previous studies of SAC/VAL in other cardiovascular diseases in experimental and clinical studies^17,25–27^. Furthermore, with the persistent MR in the MR vehicle-treated rats, the progressive LV expansion and annular dilatation can increase severity of MR suggested by El Sabbagh and colleagues^28^. Therefore, it is possible that the SAC/VAL decreased severity of MR due to less LV remodeling and subsequently less mitral annular dilatation.

The alteration of LV wall thickness after SAC/VAL administration in LV hypertrophy rats secondary to systemic hypertension and pressure overload-induced mice had also been observed^29,30^. In the current study, the CSA, an index of LV hypertrophy, was measured and demonstrated that chronic MR increased CSA while SAC/VAL attenuated LV CSA suggesting the anti-hypertrophic effect of SAC/VAL. The result of CSA in this study is also in agreement with previous studies in rats and mice^31,32^.

Compared with the MR group, 8-week treatment with SAC/VAL significantly attenuated cardiac remodeling. This beneficial effect was in line with the previously published reports in MI rats and diabetic cardiomyopathy mice^24,33^. Following the assessment of cardiac remodeling, we identified the anti-fibrotic effect as another positive impact of SAC/VAL. With our findings, SAC/VAL effectively mitigated LV fibrosis in MR-volume overload heart, which was in agreement with previous studies in diabetic cardiomyopathy mice^33^, cardiorenal syndrome-associated renal damage rats^20^, and pressure overload rats^21^. These effects were likely due to the combination of neprilysin enzyme (NEP) inhibition, which promotes and prolongs the anti-fibrotic effect of NPs in conjunction with the blockage of the fibrotic effect of Ang II through AT_1_R.

In addition, the drug potentially enhanced other downstream Ang II pathways (Ang II-AT_2_R and Ang 1–7-Mas receptor), which also provide an anti-fibrotic effect^34,35^. These advantages provided by SAC/VAL were substantiated as an insight mechanism associated with improving cardiac function. Similarly, a previous study in doxorubicin-induced dilated cardiomyopathy mice also revealed this beneficial effect of SAC/VAL^15^.

The prominent role of mitochondria in cardiomyocytes is to maintain energy homeostasis. In disease conditions, a tremendous amount of ROS is generated mainly in mitochondria, causing impairment of its infrastructure and function^36^. Mitochondrial dysfunction is frequently associated with a shortage of energy supply and cellular dyshomeostasis, leading to cardiac injury, pathological remodeling, cardiac dysfunction, and eventual cell death^37^. Supporting this notion, our present study demonstrated an augmentation of mitochondrial ROS production and the mitochondrial membrane potential alteration in MR rats compared with sham rats, suggesting worsening mitochondrial quality. Similarly, a previous study reported augmented ROS production secondary to MR^38^. The results revealed that SAC/VAL significantly limited mitochondrial ROS production and mitochondrial membrane potential depolarization and tended to decrease mitochondrial swelling caused by MR.

Moreover, SAC/VAL treatment decreased the production of mitochondrial ROS until the lower level was inconsistent with better control in mitochondrial membrane potential, suggesting that SAC/VAL maintained and enhanced cardiac mitochondrial quality. To date, the effects of SAC/VAL on mitochondrial dysfunction and ROS production were investigated and demonstrated similar beneficial effects in doxorubicin-induced dilated cardiomyopathy mice^17^, cardiorenal syndrome rats^20^, and pressure overload rats^21^, describing the drug offers essential positive effects on mitochondrial bioenergetics and protected cardiomyocytes from oxidative stress. AngII-AT_1_R pathway has been mentioned as a critical stimulation leading to ROS production and mitochondrial dysfunction in the failing heart^39^. The mitigation of mitochondrial ROS formation by SAC/VAL treatment was supposed to be working through this pathway.

In this study, we presented the effects of SAC/VAL on autophagy and mitophagy in the MR heart. Consistent with a previous report in severe MR patients^11^, relative LC3 and p62 mRNA expression, the autophagy marker, were upregulated in MR rats compared with sham to stimulate the autophagy mechanism for facilitating cell survival by eliminating damaged proteins in myocardial tissue. The significant finding was that administering SAC/VAL demonstrated downregulation of LC3 and p62 mRNA expression in MR. These findings agreed with a prior study that SAC/VAL treatment significantly reduced the LC3, Beclin-1, and Atg-5 protein expression in cardiorenal syndrome rats^20^. Therefore, autophagy may be an additional pathway affected by the drug. This study showed that PTEN-induced kinase 1 (PINK) was downregulated in MR rats, whereas the expression of Parkin mRNA, another mitophagy-related gene, did not change in MR conditions. Theoretically, PINK, Parkin, and p62 worked together under the disease conditions to stimulate the mitophagy pathway^40^. A previous study mentioned that a decrease in the phosphorylation of PINK might result from negative feedback stimulated by the overaccumulation of PINK itself in the setting of MR^41^. Compared with vehicle-treated rats, SAC/VAL treatment downregulated these mitophagy-related genes. Hence, the data denoted that SAC/VAL may partially affect the reduction in mitophagy activity.

The relative expression levels of ANP and BNP were upregulated in the MR group compared with the sham rats. Accordingly, it could be explained by data from a previous study that ANP and BNP mRNA expression was upregulated in the different stages of HF, including compensated HF and severe HF, respectively^42^. From our study, treatment with SAC/VAL downregulated ANP and BNP mRNA expression, reflecting the reduction of volume overload severity and cardiac stress, which were the essential factor-induced NPs production. A previous study demonstrated no significant difference in ANP and BNP protein expression in a rabbit model of atrial fibrillation^43^. This disparity may be due to differences in disease models and SAC/VAL dosages.

According to the results of this study, the combination of the two divergent pathways in SAC/VAL, including NEP inhibitor and AT_1_R blocker, demonstrated several beneficial effects in the setting of MR, especially mechanisms associated with mitochondrial quality and autophagy. In the part of the NEP inhibitor, SAC augments the beneficial effects of NPs composed of diuresis and natriuresis, resulting in reduced volume overload, anti-remodeling, anti-fibrosis, and counteracting actions of RAAS^44^. Simultaneously, the blockage of AT_1_R, the pathological pathway of RAAS, that is chronically overactivated during disease progression. As mentioned, it generates undesirable consequences, including cardiac fibrosis, oxidative stress generation, and mitochondrial dysfunction^5,7^. Thus, this drug could counteract these pathological consequences of the AngII-AT_1_R axis. However, two other pathways, including Ang II-AT_2_R and Angiotensin (1–7)-Mas receptor, work simultaneously to counterbalance the effect of AT_1_R and provide cardioprotective effects instead^45,46^. The drug might have an inhibitory effect on AT_1_R that opposes the deleterious effects of the Ang II-AT_1_R axis and enhances the salutary effects of these two pathways, which is supported by evidence from a previous study in hypertensive rats^30^. Further studies should be performed to prove this speculation and elucidate the effects of SAC/VAL on these two underlying mechanisms and the precise mechanism involved with autophagy supporting the cardioprotective effects of the drug in MR. Importantly, our findings conclude that SAC/VAL may have practical clinical implications for treating chronic MR and its consequence, HF.

The current study has several limitations. Firstly, although the current animal model is useful to evaluate the effect of medical therapy on cardiac remodeling induced by volume overload, it may not represent MR in humans^22^. This animal model is a primary MR model induced by making a hole on MV leaflet. In humans, MR may be a primary MR caused by abnormality of the MV apparatus (e.g., MV prolapse) or secondary MR due to either nonischemic or ischemic remodeling^28^. Secondly, this study measured mRNA expression of autophagy and mitophagy but did not measure protein level of indices of autophagy and mitophagy. The mRNA can only be used to predict the trends of protein expressions and/or functions; however, in most cases, the levels of mRNA expression were usually in agreement with the protein levels^47^. Thirdly, we are aware that mitochondrial quality is determined by several factors, including mitochondrial dynamic (fusion and fission), mitochondrial respiration, and mitophagic response. The current study found that SAC/VAL preserves mitochondrial quality related to mitophagic gene expression. Although the mitochondrial dynamic (fusion and fission), by determining mitochondrial dynamic regulated proteins (MFN, OPA, DRP-1), was not assessed in the current study, it is important to evaluate mitochondrial dynamic since it would strengthen our current findings. Further studies should be performed to determine mitochondrial dynamic.

## Materials and methods

### Animals

The experimental animal protocol was approved by the Institutional Animal Care and Use Committee of Chulalongkorn University Laboratory Animal Center (protocol number 2073021). Twenty-four healthy male Sprague–Dawley rats (*Rattus norvegicus*) were obtained from Nomura Siam International, Bangkok, Thailand. The body weights ranged from 280 to 300 g. The rats were acclimatized in the quarantine facility for 1 week and then were transferred to the standard rodent facility at the Chulalongkorn University Laboratory Animal Center. An individual ventilated cage was used for rat housing under constant environmental conditions of one 24-h light: dark cycle and temperature 22 °C ± 1 °C throughout the study. The rats were allowed to access autoclavable commercial feed and water ad libitum. All animal procedures were performed in accordance with the laboratory animal ethical principles and the Guide for the care and use of laboratory animals^48^. All reported methods are in accordance with the ARRIVE guidelines.

### Surgical induction of mitral regurgitation

MR was induced in 16 rats, whereas the other 8 rats underwent sham operation. First, rats were anesthetized using an isoflurane induction chamber. Then, an oro-endotracheal tube was placed in the trachea and connected to a rodent ventilator (Harvard Apparatus, Massachusetts, U.S.A.). The tidal volume was 2.5 mL with a respiratory rate of 75–80 breaths per minute to provide appropriate general anesthesia and maintain respiratory function. The surgical area was prepped by left thoracic fur clipping and cleaned with a sterile scrub technique using chlorhexidine scrub, chlorhexidine solution, and 70% alcohol sequentially. Thereafter, the rats were placed in right lateral recumbency. Perioperative analgesia and antibiotics composed of tramadol (12.5 mg/kg, intraperitoneal route: IP) and diluted enrofloxacin (10 mg/kg, subcutaneous route: SC) were given to alleviate surgical pain and reduce the risk of bacterial infection, respectively. A left thoracotomy was performed through the 4th and 5th intercostal spaces to approach the thoracic cavity. An Alm retractor was used to increase the working space. MR was generated using a 1-inch 20-gage needle puncture to the posterior mitral leaflet through the LV apex. Then, the needle was withdrawn, and appropriate pressure was applied to the LV to stop the bleeding and allow coagulation. Subsequently, transthoracic echocardiography was done to ensure that MR significantly existed through the right parasternal long-axis view with color mapping flow displaying a regurgitant jet area > 30% of the LA^49^. After the successful creation of MR, the thoracic wall and skin were anatomically closed with suitable suture materials. A similar surgical procedure was performed without the LV and mitral leaflet puncture for the sham operation. The recovering animals were moved to an individual recovery cage where they were provided a warm and dry environment and monitored until they presented completely normal behavior. They were administered diluted enrofloxacin (10 mg/kg, SC) and tramadol (12.5 mg/kg, SID, IP) daily for 5 days after surgery.

### The experimental procedures/pharmacological therapy

Four weeks after surgery, transthoracic echocardiography was done to assess the severity of the MR-volume overload model focusing on the presence of MR (> 30% of LA area) or LA enlargement (left atrium to the aortic root ratio; LA: Ao > 1.6) in all rats and used as baseline data. The MR rats (n = 16 rats) were randomly assigned to two groups: the MR group (drinking water; n = 8) and the sacubitril/valsartan (SAC/VAL) group (68 mg/kg PO daily; n = 8)^14^. All rats received the treatments daily via oral gavage for 8 weeks using the appropriate doses in the previous study^14^. For the sham group, the rats were given the vehicle daily (drinking water) in the same volume as other treatment groups.

### Echocardiography

Transthoracic echocardiography was done 4 weeks after surgery (baseline) and at the end of week 8 after the drug administration to evaluate drug effects on cardiac geometry and cardiac function in all groups. The Mindray M9 echocardiographic machine (Mindray, Shenzhen, China) equipped with a P10-4E (4–10 MHz) phase array transducer probe was used to acquire conventional echocardiographic parameters. The rats were mildly sedated with isoflurane, and the fur around the thoracic area was clipped. Then, the rats were placed in the right, lateral recumbent position and electrocardiographic (ECG) electrodes were attached to both forelimbs and the left hindlimb and connected to the machine.

LV conventional echocardiography consists of the interventricular septum and LV posterior wall thickness during systole and diastole (IVSd, IVSs, LVPWd, and LVPWs) and the left ventricular internal dimension end-diastole and end-systole (LVIDd, LVIDs) derived from the M-mode of the right parasternal short-axis view at the head of the papillary muscle level. From this view, the LV FS, LV EF, LV EDV, and LV ESV were calculated using the Teichholz method^50,51^. A standard two-dimensional (2D) right parasternal short-axis view at the base of the heart was performed to acquire the left atrium to aortic root ratio (LA: Ao). The right parasternal long-axis view with color mapping was performed to assess the MR jet area (%), which indicated the severity of MR^22^. In addition, the aortic flow velocity (AV Vmax) was obtained. All parameters were obtained from an average of six consecutive cardiac cycles.

### Tissue collection and fibrosis assessment

At the end of 8 weeks after drug administration, all rats were euthanized with overdose of isoflurane in an induction chamber connected to scavenging system. The heart was instantly harvested and perfused with ice-cold phosphate-buffered saline (PBS). Heart weight was obtained for the HW to BW ratio calculation. The LV-free wall was separated into three segments, similar regions, for further analysis. For the first part, approximately 30 mg of tissue was preserved in RNAlater and kept at − 20 °C for further PCR analysis. In another part, approximately 300 mg of the LV-free wall was immediately used as fresh LV tissue for mitochondrial isolation and mitochondrial quality analyses. The remaining LV section was preserved in 10% buffered formalin and embedded in paraffin. Then, myocardium-embedded blocks were sectioned and stained with hematoxylin and eosin stain (H&E) for histomorphology analyses. In addition, the blocks were sectioned into 5-µm-thick slices and stained with Masson’s trichrome stain to evaluate myocardial collagen and fibrosis. The fibrotic area was quantitatively evaluated from scanning of the whole LV section using NIS-element BR analysis software (Nikon Corporation, Tokyo, Japan): the collagen-stained area/the entire sectioned area × 100 and expressed as a percentage of the fibrotic area^52^. The left ventricular myocyte cross-sectional area was measured using NIS-element BR analysis software as previously described^31^. Briefly, each myocyte was identified, and its border was traced and the pixels within the border were calibrated against a scale to measure the area. The CSA data were averaged from 30 different cells in each image.

### Evaluation of mitochondrial quality

All processes associated with mitochondrial quality analyses were modified from previous studies^53^.

#### Mitochondrial isolation

Fresh LV tissue (approximately 300 mg) was excised and washed in ice-cold PBS solution. Then, the tissue was homogenized in ice-cold isolated buffer (300 mM sucrose, 0.2 mM EGTA, 5 mM TES, pH 7.2: 1 mL per tissue 100 mg). The homogenized tissue solution was centrifuged at 800 × g, 4 °C for 10 min. The supernatant (S1) was transferred to a precooled Eppendorf tube and re-centrifuged at 8000 × g, 4 °C for 10 min. Then, the supernatant (S2) was discarded, and the mitochondrial pellet (P2) was washed by resuspending in an ice-cold isolation buffer again and re-centrifuged at 8000 × g, 4 °C for 10 min. Finally, the isolated cardiac mitochondrial protein was resuspended with respiration buffer (100 mM KCl, 50 mM sucrose, 10 mM HEPES, and 5 mM KH_2_PO_4_, pH 7.2), and the mitochondrial protein concentration was determined using a Bradford assay (HiMedia Laboratories, Maharashtra, India) at λ 595 nm. Bovine serum albumin was used as a protein standard. The final concentration of the isolated cardiac mitochondrial stock was diluted to 0.4 mg/mL.

#### Determination of mitochondrial swelling

Mitochondrial swelling is the condition used to determine the sensitivity to membrane permeability transition (mPT)^54^. Five μL of respiration buffer and 20 mM CaCl_2_ were separately added into 2 wells of a 96-well plate, labeling them with and without calcium conditions. The isolated cardiac mitochondrial stock (200 μL) was pipetted into these two wells. The plate was measured to determine the rapid loss of the absorbance at λ 540 nm via the spectrophotometric method at 10- and 30-min time points at room temperature. The maximal mitochondrial (∆OD540 nm) swelling amplitude was calculated from the optical density at 10 min (OD1)–30 min (OD2)^55^. An increase in the ∆OD540 nm indicated mitochondrial swelling.

#### Determination of mitochondrial ROS production

Non-fluorescent 2′7′-dichlorofluorescein-diacetate (DCFH-DA), which was hydrolyzed by ROS in mitochondria and changed the form to fluorescent 2′,7′-dichlorohydrofluorescein (DCF), was used to evaluate mitochondrial ROS production^56^. The isolated cardiac mitochondrial stock was incubated with 2 μM DCFH-DA (prepared from 5 mL of respiratory buffer + 10 μL stock of 1 mM DCFH-DA) at 37 °C in the dark for 30 min. A fluorescence microplate reader with the excitation of λ 485 nm and emission of λ 530 nm was used to determine the fluorescence intensity of DCF, which indicated the increase in ROS production.

#### Determination of mitochondrial membrane potential changes

The JC-1 dye or 5,5′,6,6′-tetrachloro-1,1′,3,3′-tetraethylbenzimidazolecarbocyanine iodide (5 μM) was used in this study for an indirect estimate of the mitochondrial membrane potential change (∆ψm). Five μM of JC-1 (prepared from 5 mL of respiratory buffer + 10 μL stock of 2.5 mM JC-1) was incubated with isolated cardiac mitochondrial stock at 37 °C in the dark for 30 min. When membrane depolarization occurs (∆ψm augmentation), the expression of the red and green fluorescence is altered, leading to a decrease in the red: green ratio^54^. The red and green fluorescence intensity was measured via a fluorescence microplate reader with the excitation at λ 485 nm and emission at λ 530 nm for red and λ 590 nm for green fluorescein. The results revealed the red: green fluorescence intensity ratio.

### Real-time polymerase chain reaction (real-time PCR) analysis

The expression levels of autophagy-related genes (LC3-phosphatidylethanolamine conjugate and p62), mitophagy-related genes (PINK and Parkin), and natriuretic peptides (ANP and BNP) were determined using real-time PCR analysis. The 18S rRNA were selected and used as an internal control in this study. The used primer sequences of these genes were obtained from a previous study in rat cardiomyocytes and are displayed in Table 1^57–59^. Total RNA was extracted from the LV tissue using an AccuPure tissue RNA mini kit (AccuBioMed®, New Taipei City, Taiwan) in accordance with the manufacturer’s instructions and reverse transcribed to the first-strand complementary DNA (cDNA) using the iScript™ reverse transcription supermix (BioRad, Hercules, USA). The expression level of the target genes and housekeeping genes was detected by real-time PCR analysis using PowerUp™ SYBR™ Green Master Mix in conjunction with QuantStudio™ 5 (Thermo Fisher Scientific, Massachusetts, USA), performing duplicate per reactions. The standard reaction conditions were 95 °C for 15 s, 60 °C for 15 s, and 72 °C for 1 min with a total of 40 cycles. The threshold cycle (CT) of 18S rRNA was used to normalize the CT of the target genes (∆CT). Thereafter, the fold change mRNA expression of the target genes to the control group and the fold change mRNA expression of the target genes to the MR group was calculated using formula 2^−∆∆CT^^60^.

**Table 1:** Target genes and internal control primer sequences.

Gene	Primer sequence (5′-3′)	Base pair
p62	Forward primer: 5′-GGAACTGATGGAGTCGGATAAC-3′ Reverse primer: 5′-GGAACTGATGGAGTCGGATAAC-3′	80
LC3	Forward primer: 5′-GAGCAGCACCCCACCAAGA-3′ Reverse primer: 5′-ATTCACCAGGAGGAAGAAGGCT-3′	179
PINK	Forward primer: 5′-CCAAACACCTTGGCCTTCTA-3′ Reverse primer: 5′-CTGCCGAGATATTCCACATCAT-3′	122
Parkin	Forward primer: 5′-CCGGTGACCATGATAGTGTTT-3′ Reverse primer: 5′-CACTTCCTTGAGCTGGAAGAT-3′	95
ANP	Forward primer: 5′-GAGGAGAAGATGCCGGTAG-3′ Reverse primer: 5′-CTAGAGAGGGAGCTAAGTG-3′	93
BNP	Forward primer: 5′-TGATTCTGCTCCTGCTTTTC-3′ Reverse primer: 5′-GTGGATTGTTCGGAGACTG-3′	91
18s rRNA	Forward primer: 5′-CCGCGGTTCTATTTTGTTGGTTTT-3′ Reverse primer: 5′-CGGGCCGGGTGAGGTTTC-3′	399

### Statistical analysis

Statistical analysis was performed using IBM® SPSS® statistics commercial software (SPSS Inc., IL, U.S.A.). Continuous variables are demonstrated as mean ± standard error of mean (SEM). Student *t*-test was performed to differentiate the difference between the Sham and MR groups at baseline of the study. One-way ANOVA test with Tukey’s correction for multiple comparisons was used to evaluate the pharmacological effects of the drugs among groups. *P* value < 05 was considered statistically significance.

## Data Availability

The data that support the findings of this study are available from the corresponding author upon reasonable request. Some data may not be available because of privacy or ethical restrictions.

## Abbreviations

ANP: Atrial natriuretic peptide
AT_1_R: Angiotensin II type 1 receptor
BNP: Brain (B-type) natriuretic peptide
BW: Body wight
CSA: Cross-sectional area
EDV: End-diastolic volume
EF: Ejection fraction
ESV: End-systolic volume
FS: Fractional shortening
HF: Heart failure
HW: Heart weight
HW:BW: Heart weight per body weight ratio
LA: Left atrial
LA:Ao: Left atrial to aortic root ratio
LC3: Microtubule-associated protein 1A/1B-light chain 3
LV: Left ventricular
LVIDd: Left ventricular internal diameter diastole
LVIDs: Left ventricular internal diameter systole
MR: Mitral regurgitation
NEP: Neprilysin enzyme
NPs: Natriuretic peptide system
PINK1: PTEN-induced kinase 1
RAAS: Renin–angiotensin–aldosterone system
ROS: Reactive oxygen species
SAC/VAL: Sacubitril/valsartan
